# Inflammation as a predictor of acute kidney injury and mediator of higher mortality after acute kidney injury in non-cardiac surgery

**DOI:** 10.1038/s41598-019-56615-4

**Published:** 2019-12-30

**Authors:** Miho Murashima, Masatoshi Nishimoto, Maiko Kokubu, Takayuki Hamano, Masaru Matsui, Masahiro Eriguchi, Ken-ichi Samejima, Yasuhiro Akai, Kazuhiko Tsuruya

**Affiliations:** 1 Department of Nephrology, Nara Medical University, Nara, Japan; 2 Department of Nephrology, Nagoya City University Graduate School of Medical Sciences, 1-Kawasumi, Mizuho-cho, Mizuho-ku, Nagoya 467-8602 Japan; 3 Department of Nephrology, Nara Prefecture General Medical Center, Nara, Japan; 4 Osaka University Graduate School of Medicine, Department of Inter-Organ Communication Research in Kidney Disease, 2 Chrome-2 Yamadaoka, Suita, Osaka 565-0871 Japan

**Keywords:** Epidemiology, Acute kidney injury

## Abstract

This retrospective cohort study examined the roles of inflammation in acute kidney injury (AKI). Serum albumin and C-reactive protein (CRP) were used as markers of inflammation. Adults who underwent non–cardiac surgery from 2007 to 2011 were included. Exclusion criteria were urological surgery, obstetric surgery, missing data, and pre-operative dialysis. Subjects were followed until the end of 2015 or loss to follow-up. Associations between pre–operative albumin or CRP and post-operative AKI or association between AKI and mortality were examined by logistic or Cox regression, respectively. Mediation analyses were performed using albumin and CRP as mediators. Among 4,538 subjects, 272 developed AKI. Pre-operative albumin was independently associated with AKI (odds ratio [95% confidence interval (CI)]: 0.63 [0.48–0.83]). During a median follow-up of 4.5 years, 649 died. AKI was significantly associated with mortality (hazard ratio [HR] [95% CI]: 1.58 [1.22–2.04]). Further adjustment for pre-operative albumin and CRP attenuated the association (HR [95% CI]: 1.28 [0.99–1.67]). The proportions explained by mediating effects of lnCRP and albumin were 29.3% and 39.2% and mediation effects were statistically significant. In conclusion, inflammation is a predictor of AKI and a mediator of mortality after AKI. Interventions targeting inflammation might improve outcomes of AKI.

## Introduction

It has been consistently reported that acute kidney injury (AKI) is independently associated with higher mortality after adjusting for baseline characteristics^1–11^. However, the reasons for this association has not been fully elucidated. On the other hand, chronic kidney disease is also associated with higher mortality compared with those with normal renal function even after adjusting for baseline characteristics^12,13^. There is accumulating evidence that chronic inflammation plays important roles in worse outcomes in chronic kidney disease^14^.

The objective of this study is to examine the roles of inflammation in the development of AKI and outcomes after the event of AKI. Serum albumin and C-reactive protein (CRP) levels were used as markers of inflammation^14^. Previous studies showed that lower albumin or higher CRP levels were predictors of contrast-induced nephropathy^15–19^. However, whether serum albumin and CRP levels were associated with AKI in other clinical settings has largely been unknown. Also, whether higher mortality in those with AKI is mediated by inflammation has not been studied.

It has been reported that one-third of AKI happens post-operatively^20^. The NARA-AKI Cohort is a cohort of patients undergoing non-cardiac surgery. We hypothesized that inflammation was a predictor of AKI and a mediator of higher mortality after AKI. We tested these hypotheses on the NARA-AKI Cohort.

## Results

**Figure 1:**
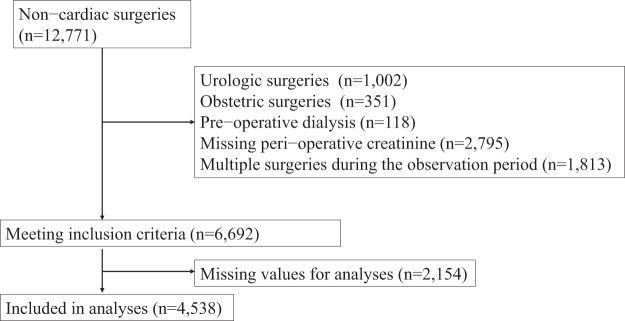
Flow of patients.

During the study period, there were 12,771 non-cardiac surgeries at Nara Medical University Hospital. After excluding subjects by exclusion criteria, data for 6,692 subjects met the inclusion criteria. After excluding subjects with missing values for analyses, data for 4,538 subjects were available for analyses (Fig. 1). Among 4,538 subjects, 272 (6.0%) developed AKI. There were only 6 cases of dialysis-requiring AKI. Demographics were shown in Table 1. There were significant differences between demographics of those with and without AKI, including higher age, higher proportion of male sex, higher proportion with comorbidity among those with AKI. Of note, those with AKI had significantly higher serum CRP levels and lower serum albumin levels.

**Table 1:** Demographics.

	No AKI (n = 4266)	AKI (n = 272)	p
Age	63 (50–72)	67 (55–74)	<0.001
Sex (male)	1995 (46.8)	159 (58.5)	<0.001
Body mass index (kg/m^2^)	22.3 (22.3–24.8)	23.4 (21.0–25.8)	<0.001
Diabetes mellitus	655 (15.4)	72 (26.5)	<0.001
Hypertension	1497 (35.1)	130 (47.8)	<0.001
Atrial fibrillation	108 (2.5)	11 (4.0)	0.17
Congestive heart failure	54 (1.3)	14 (5.1)	<0.001
Peripheral vascular disease	46 (1.1)	4 (1.5)	0.54
Ischemic stroke	235 (5.5)	13 (4.8)	0.78
Hemorrhagic stroke	97 (2.3)	10 (3.7)	0.15
Coronary artery disease	200 (4.7)	22 (8.1)	0.02
COPD	111 (2.6)	7 (2.6)	1.00
Chronic hepatitis	167 (3.9)	32 (11.8)	<0.001
Liver cirrhosis	41 (1.0)	11 (4.0)	<0.001
Malignancy	1713 (40.2)	151 (55.5)	<0.001
Smoking No Previous Current	2336 (54.8) 930 (21.8) 1000 (23.4)	135 (49.6) 80 (29.4) 57 (21.0)	0.01
CRP (mg/dL)	0.10 (0.10–0.30)	0.20 (0.10–1.20)	<0.001
Albumin (g/dL)	4.3 (4.0–4.5)	4.0 (3.5–4.3)	<0.001
Hematocrit (%)	38.2 (34.8–41.5)	35.5 (31.3–39.5)	<0.001
eGFR (mL/min/1.73m^2^)	78.1 (65.9–92.0)	69.5 (48.5–88.3)	<0.001
Dipstick proteinuria − +/− + 2+ 3+	3601 (84.4) 310 (7.3) 231 (5.4) 106 (2.5) 18 (0.4)	182 (66.9) 24 (8.8) 26 (9.6) 29 (10.7) 11 (4.0)	<0.001
ACE-Is or ARBs	803 (18.8)	86 (31.6)	<0.001
Diuretics	276 (6.5)	44 (16.2)	<0.001
Anti-platelet agents	546 (12.8)	46 (16.9)	0.06
Statins	481 (11.3)	41 (15.1)	0.06
Insulin	147 (3.4)	25 (9.2)	<0.001
Oral antidiabetics	383 (9.0)	44 (16.2)	<0.001
NSAIDs	700 (16.4)	33 (12.1)	0.07
Contrast media	231 (5.4)	21 (7.7)	0.13
Pre-operative chemotherapy	218 (5.1)	13 (4.8)	1.00
Post-operative chemotherapy	574 (13.5)	45 (16.5)	0.17
Corticosteroids	229 (5.4)	21 (7.7)	0.01
Kinds of Surgery Intra-thoracic Intra-abdominal Pelvic or major joint replacement Others	430 (10.1) 800 (18.8) 662 (15.5) 2374 (55.6)	35 (12.9) 89 (32.7) 58 (21.3) 90 (33.1)	<0.001
Surgeries not for malignancy Curative resection for malignancy Palliative resection for malignancy	2791 (65.4) 1234 (29.0) 241 (5.6)	136 (50.0) 110 (40.4) 26 (9.6)	<0.001
Emergent surgery	210 (4.9)	24 (8.8)	0.01
Intra-operative vasopressors	3482 (81.6)	236 (86.8)	0.03
Intra-operative diuretics	391 (9.2)	42 (15.4)	0.001
Intra-operative lowest systolic blood pressure (mmHg)	80 (70–85)	75 (70–80)	<0.001
Intra-operative delta systolic blood pressure (mmHg)	60 (45–80)	65 (50–80)	0.04
Intra-operative fluid balance (mL/kg)	21.2 (14.0–31.4)	25.6 (16.8–41.3)	<0.001

The data were shown as n (%) or median (interquartile range). P values were by Man-Whitney U test or Fisher’s exact test.

Delta systolic blood pressure = systolic blood pressure at the beginning of surgery – intra-operative lowest systolic blood pressure

COPD: chronic obstructive pulmonary disease, CRP: C-reactive protein, eGFR: estimated glomerular filtration rate, ACE-Is: angiotensin converting enzyme inhibitors, ARBs: angiotensin receptor blockers, NSAIDs: non-steroidal anti-inflammatory drugs.

### Associations between pre-operative CRP or albumin levels and post-operative AKI

Associations of pre-operative CRP and serum albumin levels with post-operative AKI were examined using logistic regression models (Table 2). When serum albumin was not included in the model, lnCRP was an independent predictor of post-operative AKI (adjusted odds ratio [OR] [95% confidence interval (CI)]: 1.14 [1.03–1.26]). However, when serum albumin was included in the model, lnCRP was excluded from the model by backward elimination. There was a strong correlation between serum CRP and albumin levels (Pearson correlation coefficient = 0.49, p < 0.001). The best fitted model with highest p values (p = 0.87) by Hosmer-Lemeshow test was shown in Table 2. Higher pre-operative serum albumin level was independently associated with lower incidence of post-operative AKI (adjusted OR [95% CI]: 0.63 [0.48–0.83] per 1 g/dL). Association between serum albumin and CRP levels and AKI stages were also examined. There were 161, 98, and 13 patients with stage 1, 2, and 3 AKI, respectively. As the number of patients with stage 3 was small, stage 2 and 3 were combined. Albumin was associated with more severe AKI (OR [95% CI] were 0.72 [0.52–1.01] and 0.49 [0.33–0.74] for stage 1, and stage 2 and 3, respectively). LnCRP was also associated with more severe AKI (OR [95% CI] were 1.12 (0.99–1.26) and 1.19 (1.02–1.37) for stage 1, and stage 2 and 3, respectively).

**Table 2:** Predictors of post–operative acute kidney injury.

	OR (95% CI)
Male sex	1.80 (1.35–2.41)
Body mass index (per kg/m^2^)	1.10 (1.07–1.14)
Congestive heart failure	2.10 (1.06–4.15)
Chronic hepatitis	2.29 (1.48–3.56)
Hematocrit (per 1%)	0.95 (0.92–0.98)
Dipstick proteinuria	
(−)	1 (reference)
(+/−)	1.13 (0.71–1.80)
(+)	1.40 (0.88–2.23)
(2+)	3.11 (1.92–5.02)
(3+)	5.49 (2.29–13.14)
Serum albumin (per g/dL)	0.63 (0.48–0.83)
ACE-Is/ARBs	1.33 (0.99–1.79)
Insulin	1.57 (0.97–2.54)
NSAIDs	0.62 (0.41–0.92)
Corticosteroids	1.51 (0.93–2.44)
Pre-operative chemotherapy	0.64 (0.34-1.20)
Intra-thoracic surgery	1.65 (1.04–2.62)
Intra-abdominal surgery	1.28 (0.87–1.89)
Pelvic or major joint surgery	2.14 (1.45–3.16)
Others	1 (reference)
Surgery for malignancy	1.62 (1.18–2.22)
Intra-operative diuretics	1.54 (1.02–2.31)
Intra-operative fluid balance (per mL/kg)	1.01 (1.00–1.02)

Predictors of acute kidney injury were selected by backward elimination in logistic regression models from the following variables; age, sex, body mass index, diabetes mellitus, hypertension, chronic obstructive pulmonary disease, atrial fibrillation, congestive heart failure, peripheral arterial disease, ischemic stroke, hemorrhagic stroke, coronary artery disease, history of malignancy, chronic hepatitis, liver cirrhosis, smoking status (never, previous, current), estimated glomerular filtration rate, proteinuria, hematocrit, C-reactive protein (natural log-transformed), serum albumin, pre–operative use of angiotensin converting enzyme inhibitors or angiotensin receptor blockers, diuretics, insulin, oral antidiabetics, antiplatelets, statins, chemotherapy, non-steroidal anti–inflammatory drugs, contrast media, corticosteroids, kinds of surgery (intra-thoracic, intra-abdominal, pelvic or major joint, and others), whether index surgery was for malignancy, emergent surgery, intra–operative use of vasopressors and diuretics, intra-operative lowest systolic blood pressure, intra-operative drop in systolic blood pressure (systolic blood pressure at the beginning of surgery – intra-operative lowest systolic blood pressure), and intra–operative fluid balance. The best fitted model with highest p values (p = 0.87) by Hosmer-Lemeshow test was shown. ACE-Is: angiotensin converting enzyme inhibitors, ARBs: angiotensin receptor blockers, NSAIDs: non-steroidal anti-inflammatory drugs.

### Association between post-operative AKI and mortality

**Figure 2:**
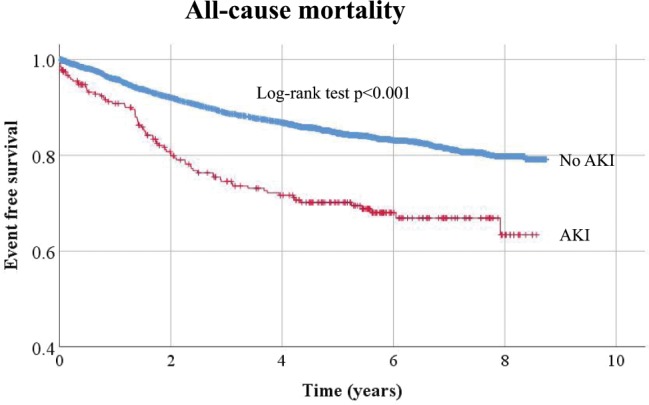
Kaplan-Meier curve for all-cause mortality among those with and without AKI. AKI: acute kidney injury.

**Figure 3:**
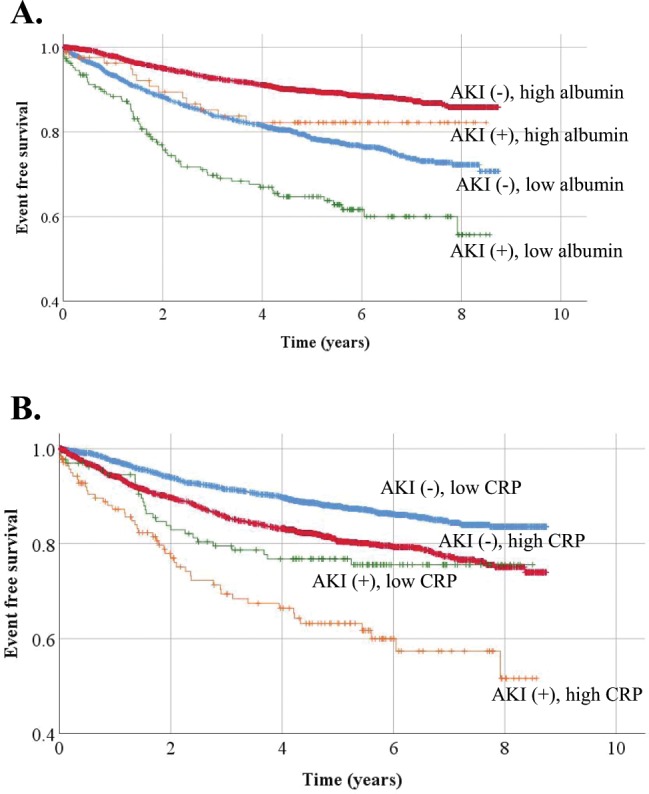
Kaplan-Meier curves for all-cause mortality stratified by AKI status and serum albumin or CRP levels. “High” represents higher than median and “low” represents lower than or equal to median. (**A**) The light blue, red, green, and orange lines represent survival curves for those without AKI and low albumin, those without AKI and high albumin, those with AKI and low albumin, and those with AKI and high albumin, respectively. (**B**) The light blue, red, green, and orange lines represent survival curves for those without AKI and low CRP, those without AKI and high CRP, those with AKI and low CRP, and those with AKI and high CRP, respectively. AKI; acute kidney injury, CRP: C-reactive proteins.

During a median follow-up of 4.5 years, there were 649 deaths (3.4 events/100 patient-years). The causes of deaths were as follows: cardiovascular death 32 (4.9%), death due to infection 43 (6.6%), death due to malignancy 480 (74.0%), and others 94 (14.5%). Kaplan-Meier curves for all-cause mortality for those with and without AKI were shown in Fig. 2. The survival curves diverged immediately post-operatively. Patients were divided into 4 groups by AKI status and albumin or CRP levels (higher than median or lower than or equal to median) and their survival curves were compared. Patients without AKI but low albumin or high CRP showed similar survival to those with AKI and high albumin or low CRP (Fig. 3). Post-operative AKI was associated with higher mortality after adjustment for potential confounders (adjusted hazard ratio [HR] [95% CI]: 1.58 [1.22–2.04]) (Table 3). With the number of patients with AKI of 272, the number of patients without AKI of 4266, α value of 0.05, HR 0.8 for mortality in those without AKI (those with AKI as a reference), and median survival of those without AKI of 4.53 years, the statistical power was 0.81. When the model was further adjusted for pre-operative lnCRP and serum albumin levels, the association between post-operative AKI and mortality was attenuated and was not significant anymore (adjusted HR [95% CI]: 1.28 [0.99–1.67]) (Table 3). Of note, there were no missing values for albumin and CRP levels and the attenuation of the association was not due to the lack of statistical power. The association between AKI and short-term mortality (mortality within 6 months) and impact of baseline inflammation on this association was similar. AKI was significantly associated with mortality (HR [95% CI]: 2.07 [1.11–3.85]) and the association was attenuated by further adjustment for lnCRP and albumin (HR [95% CI]: 1.62 [0.86–3.03] and 1.36 [0.72–2.56], respectively). There were 23 patients (1.3/1000 patient-year) and 17 patients (16.6/1000 patient-year) who developed end-stage renal disease (ESRD) among those without and with AKI, respectively. Additional adjustment for ESRD in Cox regression models did not significantly change the results. AKI was still significantly associated with mortality (HR [95% CI)]: 1.52 [1.17–1.97]). Further adjustment with ln CRP and albumin levels attenuated the association between AKI and mortality (HR [95% CI]: 1.34 [1.02–1.74] and 1.24 [0.95–1.62], respectively).

**Table 3:** Association between acute kidney injury and mortality.

	HR (95% CI)
Model 1	1.58 (1.22–2.04)
Model 1 + lnCRP	1.40 (1.08–1.81)
Model 1 + lnCRP + serum albumin	1.28 (0.99–1.67)

Data were adjusted for age, sex, smoking status (never, previous, current), body mass index, diabetes mellitus, hypertension, chronic obstructive pulmonary disease, atrial fibrillation, congestive heart failure, peripheral arterial disease, ischemic stroke, hemorrhagic stroke, coronary artery disease, history of malignancy, whether index surgery was for malignancy (not for malignancy, curative resection, palliative resection), chronic hepatitis, liver cirrhosis, estimated glomerular filtration rate, proteinuria, the use of angiotensin converting enzyme inhibitors or angiotensin receptor blocker, diuretics, insulin, oral antidiabetics, anti-platelet agents, statin, corticosteroids, pre-operative chemotherapy, or post-operative chemotherapy. CRP: C-reactive protein.

**Figure 4:**
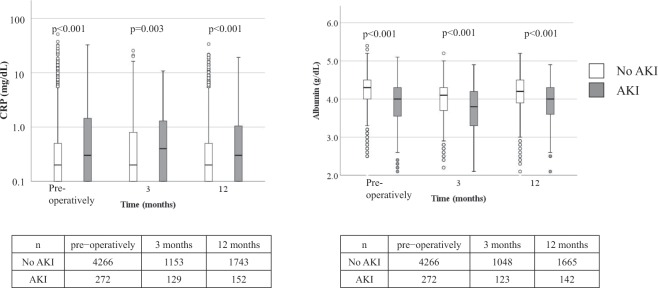
Box plots of serum CRP and albumin levels among those with and without AKI. A box represents interquartile range and a horizontal line in the box shows the median value. A bar shows range and outliers are shown in circles. P values were by Man-Whitney U test comparing values for those with and without AKI at each time point. The number of patients with available values was shown in tables below the graphs. CRP: C-reactive protein, AKI: acute kidney injury.

By mediation analyses, regression coefficients (95% CI) for pure indirect effect (mediation effect) were 0.09 (0.05–0.14) (p < 0.001) and 0.16 (0.10–0.23) (p < 0.001) for lnCRP and albumin levels, respectively. The proportion explained by mediating effect of lnCRP and albumin was 29.3% and 39.2%, respectively. CRP levels were persistently higher and serum albumin levels were persistently lower among those with AKI pre- and post-operatively, compared with those without AKI (Fig. 4).

## Discussion

In this study, the association between inflammation and post-operative AKI or mortality after AKI was examined using serum CRP and albumin levels as markers of inflammation. Lower serum albumin was independently associated with post-operative AKI. Post-operative AKI was associated with higher mortality and this association was at least partially mediated by pre-operative serum CRP and albumin levels. These results suggest that those with underlying inflammation are more likely to develop AKI and rather than AKI itself, but underlying inflammation is associated with higher mortality among those with AKI.

First, we showed that hypoalbuminemia was independently associated with development of post-operative AKI. Association between higher CRP or lower albumin and AKI have been reported in contrast-induced nephropathy^15–19^ or post-operative AKI in cardiac surgery^21,22^. In many of these studies, higher CRP was shown to be associated with AKI but the data were not adjusted for serum albumin level. One study on 1182 subjects undergoing off-pump coronary artery bypass grafting showed that serum albumin level <4.0 g/dL was associated with higher incidence of AKI (OR [95% CI]: 1.83 [1.27–2.64])^22^. Interestingly, CRP level was included in the covariates but excluded from the model by backward elimination. In another study on subjects with acute coronary syndrome undergoing percutaneous coronary intervention, higher CRP and lower serum albumin levels were significantly associated with contrast-induced nephropathy in univariate analyses. However, in multivariate analysis, only lower serum albumin level was significantly associated with contrast-induced nephropathy and association between CRP levels and contrast-induced nephropathy was not significant anymore^18^. These results are consistent with our results showing that CRP levels were significantly associated with AKI when serum albumin was not included in covariates, but CRP is excluded from the model when serum albumin level was included in the model, and that serum albumin levels, but not CRP levels, were independently associated with AKI. It is unclear why albumin, but not CRP, was independently associated with development of AKI. There is a possibility that intravascular volume contraction due to lower oncotic pressure associated with hypoalbuminemia leads to AKI. However, the odds ratio for AKI associated with serum albumin level did not change in logistic regression models after additional adjustment for lowest systolic blood pressure during surgery, maximum decline in systemic blood pressure during surgery. Although blood pressure is not a direct measure of intravascular volume, the result makes it less likely that intravascular volume contraction is the explanation for association between hypoalbuminemia and AKI.

Second, in this study, we showed that association between AKI and higher mortality was at least partially mediated through pre-operative higher CRP levels and lower serum albumin levels. There have been multiple previous studies which consistently reported association between AKI and higher mortality even after adjusting for baseline characteristics in various clinical settings^1–11^. However, the data were not adjusted for baseline serum CRP levels or albumin levels in any of these studies. Although higher CRP and lower albumin have been shown to be associated with higher mortality in a variety of clinical settings including those with AKI^23^, it has not been tested if the higher mortality among those with AKI is mediated by chronic inflammation. In our study, serum albumin was persistently lower and CRP levels were persistently higher before and after the development of AKI for those with AKI compared with those without AKI. AKI was significantly associated with mortality after adjusting for demographics and comorbidities, but further adjustment for pre-operative CRP and albumin levels attenuated the association between AKI and mortality. The mediation effect was statistically significant for both CRP and albumin levels. These data suggest that underlying chronic inflammation was a mediator of higher mortality associated with AKI.

This study has several strengths. This study was the first to suggest that association between AKI and higher mortality was at least partially mediated through underlying chronic inflammation and catabolic state. The data were vigorously adjusted for other potential confounding factors compared with other previous studies.

There are limitations of our study. As this is an observational study, there might be unknown confounding factors. The NARA-AKI Cohort is a cohort of patients undergoing non-cardiac surgery and the proportion of patients with cardiac comorbidity was low, and the proportion of patients with malignancy was high (35.5% of patients had surgery for malignancy). There is a possibility that higher CRP and lower albumin levels were reflection of the extent of malignancy, although we adjusted the data for the presence or absence of malignancy, or whether patients undergo surgery for malignancy (not for malignancy, curative or palliative resection for malignancy). As the characteristics of cohorts were different from other cohorts of AKI such as in cardiac surgery, critical care, or percutaneous coronary intervention, the generalizability of our findings requires further research. A significant number of patients had missing values for creatinine measurements perioperatively. Those patients were likely to be judged by physicians to be at low risk for AKI. Population studied in this study might be a selective population with relatively high risk for AKI among those undergoing non-cardiac surgery.

In conclusion, lower serum albumin and higher CRP levels, which are potential markers for inflammation, were significantly associated with AKI and our results suggest that higher mortality after AKI was at least partly mediated by underlying inflammation in our cohort of non-cardiac surgery. The results were hypothesis-generating and if the concept is proved, interventions targeting inflammation might prevent AKI or improve outcomes of AKI.

## Methods

### Settings and patients

This is a single center, retrospective observational study. Inclusion criteria were adult patients (age ≥ 18) who underwent non-cardiac surgery under general anesthesia from April 2007 (when electronic medical records started at our hospital) to December 2011 at Nara Medical University Hospital. Exclusion criteria were those who underwent urological surgery (as changes in creatinine due to nephrectomy or ureteral manipulation are likely to be of different mechanisms from mechanisms of other post-operative AKI), obstetric surgery, those without creatinine values within 1 month pre-operatively or 1 week post-operatively, and those who had undergone dialysis pre-operatively. If subjects underwent multiple surgeries during the study period, the first eligible surgery was considered. We further excluded those with missing values for analyses including serum albumin and CRP levels. Thus, the final dataset used for analyses did not include any missing data. The end of observation period was at the end of 2015 or loss to follow-up.

Nara Medical University Ethics Committee approved the study protocol and waived the need for informed consent as part of the study approval (Approval No. 1208 and No. 1208–2 for amendment). This study waived the requirement for written informed consent due to the retrospective nature of this study. Rather, research content has been included on the web page of our department (http://nephrology.naramed-u.ac.jp/research/clinical.html). The study was conducted in accordance with Declaration of Helsinki. The study was registered in the University hospital Medical Information Network (UMIN000037141).

### Data acquisition and definitions

The list of subjects who underwent non-cardiac surgery under general anesthesia, age, sex, date of surgery, and laboratory data were automatically abstracted from electronic medical records. Comorbidities, use of medications, and outcomes were hand-searched from electronic medical records by investigators.

AKI was defined by the KDIGO criteria^24^. Pre-operative laboratory data were defined as those within 1 month pre-operatively and the closest to the date of surgery. Estimated glomerular filtration rate (eGFR) was calculated using the equation developed for Japanese population by Japanese Society of Nephrology^25^. Serum albumin and CRP levels at 3 months and 1 year post-operatively were defined as those values at 3 months + /− 15 days and 1 year + /− 3 months, respectively.

### Statistical analyses

Data were shown as number (%) or median (interquartile range). Demographics for those with and without AKI were compared by Fisher’s exact test, or Man-Whitney U test. Association between serum albumin or CRP (natural log-transformed) levels and post-operative AKI was analyzed using logistic regression models and adjusted for the following variables; age, sex, body mass index, diabetes mellitus, hypertension, chronic obstructive pulmonary disease, atrial fibrillation, congestive heart failure, peripheral arterial disease, ischemic stroke, hemorrhagic stroke, coronary artery disease, history of malignancy, chronic hepatitis, liver cirrhosis, smoking status (never, previous, current), eGFR, proteinuria, hematocrit, CRP (natural log-transformed), serum albumin, pre-operative use of angiotensin converting enzyme inhibitors or angiotensin receptor blockers, diuretics, insulin, oral antidiabetics, antiplatelets, statins, chemotherapy, non-steroidal anti-inflammatory drugs, contrast media, and corticosteroids, kinds of surgery (intra-thoracic, intra-abdominal, pelvic or major joint, and others), whether index surgery was for malignancy (not for malignancy, curative resection, and palliative resection), emergent surgery, intra-operative use of vasopressors and diuretics, intra-operative lowest systolic blood pressure, intra-operative drop in systolic blood pressure (systolic blood pressure at the beginning of surgery – intra-operative lowest systolic blood pressure), and intra-operative fluid balance. Predictors of post-operative AKI were selected by backward elimination algorithm and the best fitted model with highest p values by Hosmer-Lemeshow test was selected. Association between serum albumin or CRP levels and AKI stages were examined using multinomial logistic regression as proportional assumption was not satisfied in ordinary logistic regression. The data were adjusted for the same covariates as shown in Table 2. Association between albumin and stages of AKI and association between lnCRP and stages of AKI were tested in separate models. Association between post-operative AKI and mortality was examined by Kaplan-Meier curves and Cox regression models and adjusted for the following variables; age, sex, smoking status (never, previous, current), body mass index, diabetes mellitus, hypertension, chronic obstructive pulmonary disease, atrial fibrillation, congestive heart failure, peripheral arterial disease, ischemic stroke, hemorrhagic stroke, coronary artery disease, history of malignancy, whether index surgery was for malignancy (not for malignancy, curative resection, palliative resection), chronic hepatitis, liver cirrhosis, eGFR, the use of angiotensin converting enzyme inhibitors or angiotensin receptor blockers, diuretics, insulin, oral antidiabetics, anti-platelet agents, statins, corticosteroids, pre-operative chemotherapy, or post-operative chemotherapy. The data were further adjusted for serum CRP (natural log-transformed) and albumin levels to see if these were confounders for association between AKI and mortality. Mediation analyses were also performed according to a method described by Discacciati *et al*.^26^. Statistical analyses were performed using SPSS version 25.0 (IBM Corp., Armonk, NY), and STATA MP version 15.1 (Stata Corp., College Station, TX). Power calculation was performed by Power and Sample Size Program version 3.1.6 (Department of Biostatistics, Vanderbilt University Medical Center, Nashville, TN).

### Ethical approval and informed consent

Nara Medical University Ethics Committee approved the study protocol and waived the need for informed consent as part of the study approval (Approval No. 1208 and No. 1208-2 for amendment). This study waived the requirement for written informed consent due to the retrospective nature of this study. Rather, research content has been included on the web page of our department (http://nephrology.naramed-u.ac.jp/research/clinical.html). The study was conducted in accordance with Declaration of Helsinki. The study was registered in the University hospital Medical Information Network (UMIN000037141).

## Data Availability

The datasets generated during and/or analyzed during the current study are available from the corresponding author on reasonable request.
